# Pregnancy incidence and outcomes in women with perinatal HIV infection

**DOI:** 10.1097/QAD.0000000000001552

**Published:** 2017-07-12

**Authors:** Laura Byrne, Rebecca Sconza, Caroline Foster, Pat A. Tookey, Mario Cortina-Borja, Claire Thorne

**Affiliations:** a UCL Great Ormond Street Institute of Child Health; b Imperial College Healthcare NHS Trust, London, UK.

**Keywords:** antiretroviral agents, HIV, incidence, paediatrics, perinatal, pregnancy outcome, pregnant women, viral load

## Abstract

**Objectives:**

To estimate the incidence of first pregnancy in women living with perinatally acquired HIV (PHIV) in the United Kingdom and to compare pregnancy management and outcomes with age-matched women with behaviourally acquired HIV (BHIV).

**Design:**

The National Study of HIV in Pregnancy and Childhood is a comprehensive, population-based surveillance study that collects demographic and clinical data on all pregnant women living with HIV, their children, and all HIV-infected children in the United Kingdom and Ireland.

**Methods:**

The incident rate ratio of first pregnancy was calculated for all women of reproductive age who had been reported to the National Study of HIV in Pregnancy and Childhood as vertically infected children. These women and their pregnancies were compared to age-matched pregnant women with BHIV.

**Results:**

Of the 630 women with PHIV reported in the United Kingdom as children, 7% (45) went on to have at least one pregnancy, with 70 pregnancies reported. The incident rate ratio of first pregnancy was 13/1000 woman-years. The BHIV comparison group comprised 118 women (184 pregnancies). Women with PHIV were more likely to be on combined antiretroviral therapy at conception and have a lower baseline CD4^+^ cell count (*P* < 0.01 for both). In adjusted analysis, PHIV and a low baseline CD4^+^ cell count were risk factors for detectable viral load near delivery; older age at conception and being on combined antiretroviral therapy at conception reduced this risk.

**Conclusion:**

Women with PHIV in the United Kingdom have a low pregnancy incidence, but those who become pregnant are at risk of detectable viral load near delivery, reflecting their often complex clinical history, adherence, and drug resistance issues.

## Introduction

Mother-to-child transmission (MTCT) of HIV peaked worldwide in 2001-2002 and the estimated number of annual new infections has subsequently fallen by 52% [1]. Following advances in treatment with combined antiretroviral therapy (cART) and the associated increases in life expectancy [2], the first generation of young people with perinatally acquired HIV (PHIV) has now reached adulthood [3]. The emphasis of care for young people living with PHIV has therefore shifted to transition of care to adult services, continued management of adherence, complex resistance, long-term drug toxicity, and sexual, reproductive, and mental health [4,5].

In the United Kingdom, approximately 1200 pregnancies in women living with HIV are currently reported annually, with the MTCT rate at an all-time low of 0.27% [6]; an increasing number of pregnancies are now in women with PHIV.

Several studies in resource-rich settings have investigated pregnancy rates and outcomes in women with PHIV [7–14], reporting lower pregnancy incidence rates compared with HIV-negative women [7,9]. Half of the pregnant women living with PHIV in an earlier UK case series had adherence problems [12] and US studies have shown higher HIV viral load during pregnancy but similar rates of MTCT compared with women with behaviourally acquired HIV (BHIV) [8,9,13]. Being born to a woman with PHIV was an independent risk factor for poor fetal and infant growth when compared to being born to a woman with BHIV [11,15]. Several studies have compared women with PHIV to those with BHIV to estimate the effects of mode of HIV acquisition on pregnancy and infant outcomes [7,8,11,13]; however, such comparisons have been limited by key differences between groups regarding age, parity, and treatment era.

There is growing interest in the health of HIV-exposed but uninfected (HEU) children as they have worse health outcomes than HIV-unexposed infants [16]. HEU infants born to women with PHIV may be particularly affected as their mothers are more likely to have low CD4^+^ cell counts, detectable viraemia, and AIDS-related morbidity, as well as the hitherto unknown effects of maternal immune dysfunction caused by lifelong HIV infection on the developing foetus. Additionally, there are concerns regarding potential dysfunction of inherited mitochondria in the foetus because of early childhood ART exposure of women with PHIV.

Our aims were to estimate the incidence of first pregnancy in women living with PHIV in the United Kingdom and Ireland using national surveillance data and to compare their pregnancy management and outcomes with those in an age-matched group of women with BHIV, with a focus on delivery with detectable viral load and adverse pregnancy outcomes.

## Methods

In the United Kingdom and Ireland, data are collected on pregnant women living with HIV and their HIV-exposed children and on all HIV-infected children through a comprehensive, population-based surveillance study, the National Study of HIV in Pregnancy and Childhood (NSHPC); pregnancies in women with diagnosed HIV are notified through a quarterly active surveillance scheme regardless of outcome using standardized reporting forms from all maternity units [17,18]. Children are classified as vertically infected if reported with known exposure to maternal HIV infection. The dataset used included all diagnosed women with at least one pregnancy reported to the NSHPC by September 2014.

The NSHPC has London multi-centre research ethics committee approval (MREC/04/2/009).

## Estimating incidence rates of first pregnancy reported in women with perinatally acquired HIV

The population considered ’at-risk’ of pregnancy were all women with PHIV ever reported to the NSHPC as children who had not died or gone abroad before their 13th birthday. Time ’at-risk’ of first pregnancy was considered to be from their 13th birthday to 30th June 2014 ('study end date’) or date of censoring. Time to estimated conception date (estimated delivery date minus 280 days for ongoing pregnancies, actual delivery date minus length of gestation for other outcomes) of first pregnancy was calculated for reported pregnancies. Individuals without a reported pregnancy were censored at date of death or date of last contact with UK health services if lost to follow-up or known to have gone abroad before study end date. The incidence rate of first pregnancy was also calculated for women with PHIV aged 16–24 years, with time ’at risk’ of first pregnancy calculated from their 16th to 25th birthday, or date of death or last contact as applicable if this was earlier.

## Comparing pregnancy management and outcomes of women living with perinatally acquired HIV and behaviourally acquired HIV

Eligibility criteria for inclusion in the pregnant women with PHIV group were: at least one reported pregnancy; reported as vertically infected with no other risk reported; diagnosis before their 14th birthday. Forty-five women met these criteria and comprised the PHIV group; years of delivery for their pregnancies spanned 2006–2014.

To create an age-matched BHIV pregnant women group, women were considered for inclusion if they met the following criteria: first pregnancy reported with estimated or actual delivery date in or after January 2006 (to align with the time period of the deliveries to PHIV women); nulliparous at first reported pregnancy (or missing parity at first reported pregnancy with no indication of any previous pregnancy); diagnosed before their first reported pregnancy; not reported as vertically infected; diagnosed after their 13th birthday if mode of acquisition was known or after their 15th birthday if this was unknown; aged 29 or less years at estimated conception of first reported pregnancy. There were 943 women eligible for the BHIV group.

Eligible women for both groups were then classified according to age at first reported conception (<16, 16–19, 20–24, 25–29 years). In age bands with excess numbers of eligible BHIV women, BHIV women were retained randomly to achieve the target of three times the number of PHIV women. Forty-five women were in the PHIV pregnant women group and the final, age-matched BHIV comparison group comprised 118 women.

### Definitions

The first trimester was defined as less than 13 completed gestational weeks; second as 13–26 weeks; and third, at least 27 weeks.

Baseline CD4^+^ T-cell counts (cells/μl) were the earliest reported measurements during pregnancy, CD4^+^ T-cell counts and HIV viral load measurements (copies/ml) at delivery were those taken closest to delivery, either during the third trimester or within seven days after delivery. Undetectable viral load was defined as an HIV RNA measurement less than 50 copies/ml.

Caesarean sections taking place before rupture of membranes and/or onset of labour were classified as elective. Deliveries before 37 completed gestational weeks were considered preterm. Low birth weight (LBW) was defined as less than 2500 g [19]. Infant HIV infection status was classified as uninfected or infected based on reported PCR or HIV antibody results or indeterminate if infection status had not yet been reported or confirmed.

Congenital abnormalities were classified according to the WHO international classification of diseases, 10th revision, using information provided by clinicians at infant notification and/or follow-up.

### Statistical analysis

Proportions calculated among cases were compared using the χ^2^ or Fisher's exact test; trends in proportions were assessed using the χ^2^ test for trend [20]. For nonnormally distributed variables, medians were compared using the Wilcoxon-Mann-Whitney test [20,21] and trends in medians using Cuzick's [22] nonparametric test for trend across ordered groups.

Logistic regression models were fitted to calculate estimates of odds ratios and adjusted odds ratios to examine factors associated with detectable viral load near delivery among pregnancies ending in live birth.

To account for clustering of data at the woman level (some contributed more than one pregnancy), robust standard errors were calculated using a clustered sandwich estimator [23]. Multivariable models were developed using a forward fitting selection strategy. Multiple pregnancies ending in live births were treated as a single event, but the total number of infants born was included when ’infant’ is used. Potential collinearity between explanatory variables was examined by calculating variance inflation for the regression model estimates.

Data were managed in Access 2010 (Microsoft Corp., Redmond, Washington, USA), compiled using R version 2.14.2 [24], and analysed using Stata version 13 (Stata Corp. LP, College Station, Texas, USA).

## Results

### Incidence of first reported pregnancy in women with perinatally acquired HIV

There were 630 women reported to the NSHPC in childhood with PHIV, with total follow-up time of 3568 woman-years; 45 (7%) had at least one pregnancy reported. The incidence rate of first pregnancy was 13 per 1000 woman-years [95% confidence interval (CI): 9–17 per 1000 woman-years]. Age at first estimated conception date ranged from 13 to 27 years. The incidence rate of first pregnancy in the subgroup of 470 PHIV women aged 16–24 years was 22 per 1000 woman-years (95% CI: 16–30 per 1000 woman-years), with a total follow-up time of 1911 woman-years.

### Comparing pregnancy management and outcomes between perinatally acquired HIV and behaviourally acquired HIV groups

**Fig. 1:**
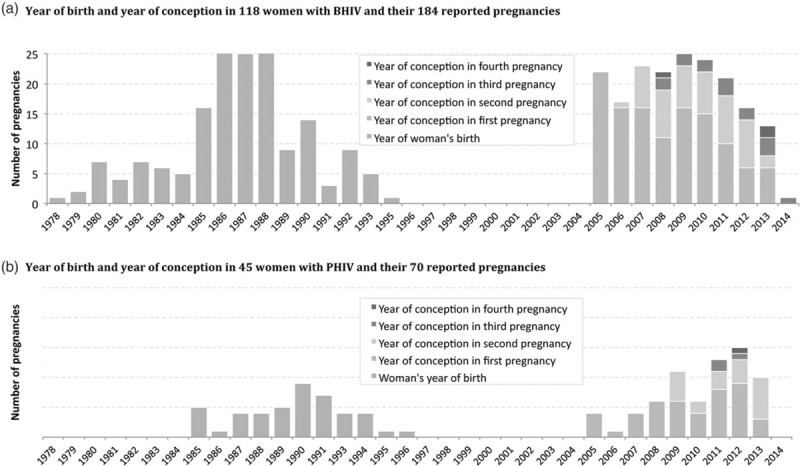
(a) Year of birth and year of conception in 118 women with BHIV and their 184 reported pregnancies. (b) Year of birth and year of conception in 45 women with PHIV and their 70 reported pregnancies.

BHIV, behaviourally acquired HIV; PHIV, perinatally acquired HIV.

The 45 women with PHIV had 70 pregnancies reported (21 second pregnancies, three third pregnancies, and one fourth pregnancy; all singleton). The age-matched BHIV comparison group of 118 women had 184 pregnancies [48 second pregnancies, 15 third pregnancies, and three fourth pregnancies; (one twin pregnancy with concordant outcomes)]. Women with PHIV were more likely to be born in the United Kingdom, younger at first conception, and more likely to be living in London than women with BHIV (Table 1). Year of birth for the women and year of conception for their reported pregnancies are shown in Fig. 1a and b.

In most pregnancies, the women received antenatal cART, though more pregnancies in women with PHIV were conceived on cART and had a lower baseline maternal CD4^+^ cell count in pregnancy (Table 2). Median gestation at initiation of cART in pregnancies not conceived on cART was 21 weeks (interquartile range: 17–24 weeks) in women with BHIV and 17 weeks in women with PHIV (interquartile range: 10–24 weeks; *P* = 0.03). Class of cART prescribed did not significantly differ between the two groups amongst live births and continuing pregnancies: in 14% (8/59) of pregnancies in women with PHIV (‘PHIV pregnancies’) and 26% (42/161) of those in women with BHIV (‘BHIV pregnancies’) nonnucleoside reverse transcriptase inhibitor (NNRTI)-based ART was given; protease inhibitor-based cART was given in 80% (47/59) of PHIV pregnancies, and 67% (109/161) of BHIV pregnancies (*P* = 0.18). Drug class differed significantly when restricted to pregnancies not conceived on cART: NNRTI-based cART was started in 18% (7/39) of PHIV pregnancies and 48% (32/67) of BHIV pregnancies; in 74% (29/39) of PHIV pregnancies and in 42% (28/67) of BHIV pregnancies, women started protease inhibitor-based cART (*P* = 0.004). Overall, in 49% (23/47) of PHIV pregnancies with protease inhibitor-based cART, women received ritonavir-boosted darunavir, whereas for BHIV pregnancies with protease inhibitor-based regimens, ritonavir-boosted lopinavir predominated (61%, 66/109). Raltegravir was used significantly more frequently in PHIV pregnancies ending in live births or continuing than in BHIV pregnancies: 27% (16/59) versus 0.6% (1/162), respectively (*P* < 0.001).

A higher proportion of PHIV pregnancies ended in termination, whereas more BHIV pregnancies ended in miscarriage (13 versus 3%; Table 2). Among live births, there was no evidence of a difference in mode of delivery between the two groups or in the proportion of preterm deliveries or of LBW in term infants (Table 2).

### Examining the association between mode of maternal HIV acquisition and detectable viral load near delivery in live births

Of those delivering a live-born infant, 20% of women in the BHIV group and 40% of those in the PHIV group had detectable viral load near delivery (Table 3). Women with PHIV with a live birth had significantly greater odds of detectable delivery viral load in univariable analysis than women with BHIV (*P* = 0.03; Table 3); age at first conception, age at conception in current pregnancy, cART at conception, baseline CD4^+^ cell count less than 200 cells/μl, and protease inhibitor-containing ART were also associated with detectable viral load.

In the multivariable final model, the significant association with maternal mode of acquisition remained, with PHIV pregnancies having 3.22 times higher odds of detectable viral load near delivery (Table 3). Receipt of a protease inhibitor-based regimen was also associated with increased odds of unsuppressed viral load, whereas conception on cART was associated with significantly lower odds. There was an indication that maternal CD4^+^ cell count of less than 200 cells/μl was associated with an increased odds of detectable viral load (adjusted odds ratio = 3.39, *P* = 0.05).

### Infant outcomes

In the BHIV group, 1.9% (95% CI: 0.34–5.4, 3/160 infants) infants had a major congenital abnormality versus 5.7% (95% CI: 1.2–15.7, 3/53) in those born to women with PHIV (*P* = 0.15). Three of the six infants with defects had first trimester exposure to ART (two from conception and one from 11 weeks gestation).

Overall, only one infant was known to be diagnosed with HIV (positive PCR at age ≤72 h), whose mother with PHIV had longstanding adherence issues; 96% (157/163) of infants born to women with BHIV were reported as uninfected compared to 97% (56/59) of infants born to women with PHIV. Infant HIV status was not yet reported to the study in the remaining 4 and 3% of cases, respectively.

## Discussion

We report an incidence rate of first pregnancy in women with PHIV aged at least13 of 13 per 1000 woman-years and 22 per 1000 woman-years when restricted to women aged 16–24 years. The NSHPC is a longstanding national surveillance study to which all pregnancies to women diagnosed with HIV and all children exposed to and diagnosed with HIV in the United Kingdom and Ireland are reported. In the United Kingdom, antenatal HIV screening coverage exceeds 95% and reporting to the NSHPC is embedded within NHS commissioned antenatal services. We were therefore able to estimate a national incidence rate of first pregnancy in young women with PHIV for the first time. We identified some significant differences between pregnancies in women with PHIV and those in age-matched women with BHIV, including more conceptions on cART, lower CD4^+^ cell counts, and more terminations in the PHIV group. We also found that maternal PHIV was an independent risk factor for detectable delivery viral load, associated with a three-fold increased risk.

Our methodology, whereby our study population of women with PHIV had first been reported to the NSHPC in childhood with prospective follow-up, ensures confidence about reported mode of acquisition, which can be subject to recall bias. Our estimated first pregnancy incidence rate is lower than that reported in two US studies, which found incidence rates of 19 and 53 pregnancies per 1000 woman-years in women with PHIV aged 13 and above [7,9]. Our rate here is also low compared with the conception rate in England and Wales among similar age groups, which was 41 per 1000 in women aged under 20 and 96 per 1000 in those aged 20–24 years in 2013 [25].

Conception rates are influenced by sexual activity, procreational intent, fertility, and access to and use of contraception. Prevalence of sexual activity and risk-taking behaviour in young people with PHIV varies by setting. There have been several studies in the USA: one multicentre cohort study found lower rates of sexual activity in young people with PHIV compared with those with BHIV, with sexual activity more likely in those older, those with a boyfriend or girlfriend, and those using illicit drugs [26]; however, a smaller single-centre study found similar sexual risk behaviours in young people with PHIV and those with BHIV [27], whereas another cohort comparing young people with PHIV to those HEU found a lower proportion sexually active in the former, but this did not reach statistical significance [28]. In the United Kingdom, one single-centre cohort reported that 78% of young people with PHIV aged 16–25 were sexually active [29], and the adolescents and adults living with perinatal HIV cohort reported lower sexual activity in adolescents with PHIV compared with HIV-negative adolescents [30]. A qualitative study of young people with PHIV showed a high rate of procreational intent, likely reflecting perceived improvements in HIV treatment and MTCT risk [31]. A small, single-centre cohort of young women with PHIV found that 14% had a diagnosis of infertility [32]. Therefore, lower incidence of first pregnancy in women with PHIV compared to women of a similar age in the general UK population may be multifactorial: lower rates of sexual activity and lower fertility because of lifelong HIV and its associated morbidity.

However, although the proportion of live births was similar across groups, there were more reported terminations in women with PHIV, potentially reflecting differences in clinical care: women with PHIV may access care more often as they are more likely to have had AIDS-related morbidity, drug resistance, and adherence issues [12], so pregnancies ending in early termination may be better ascertained and reported by our respondents than similar pregnancies in women with BHIV. This difference could also reflect lower fertility desires and/or a higher unmet need for contraception. Reduction of unintended pregnancies through provision of contraception and reproductive health services is a key strategy in preventing perinatal HIV in resource-rich and resource-poor settings [33,34]. A higher proportion of terminations in the PHIV group indicate potential need for more closely integrated family planning services. As women with PHIV may experience fragmentation of care during transition from paediatric to adult services, it is critical to ensure their reproductive health needs are met throughout this period and across healthcare settings.

Women with PHIV were more likely to conceive on cART, reflecting their childhood diagnosis and treatment experience. Gestation at cART initiation in those untreated at conception was earlier in women with PHIV than women with BHIV (median 17 and 21 weeks, respectively); despite no difference in gestation at antenatal booking (all women had been diagnosed with HIV before their first pregnancy). This may have been because women with PHIV are more strongly linked to HIV care, or because their physicians anticipated slow viral load decay or suboptimal adherence.

Young people with PHIV have higher risk of treatment failure and multiclass drug resistance than those with BHIV for many reasons: previous exposure to obsolete and suboptimal ART; the limited range of ART licensed for paediatric use; difficulties with adherence because of stigma, discrimination, and HIV-associated neurocognitive deficits, among others [35]. These factors may be implicated in the association we and others report between having PHIV and failing to achieve undetectable viral load by delivery [8,13]. Though we do not report on treatment history or resistance, our finding of more raltegravir use among the PHIV group is suggestive of more complex treatment profiles or higher viral load in pregnancy in this group. We also found that older age and conception on cART were associated with reduced risk of detectable viral load near delivery overall, consistent with findings from elsewhere [36,37].

We found no significant difference in preterm delivery or LBW in term infants between PHIV and BHIV groups. In a US study, women with PHIV had a nearly six-fold increased risk of having a small for gestational age infant compared with BHIV women [15] and the same research group found that infants born to women with PHIV had shorter length-for-age 1 year after birth [11]. This inconsistency may be because our BHIV comparison group was restricted to women diagnosed before conception who were nulliparous at first included pregnancy; the US studies did not account for differences in gravidity or comment on timing of diagnosis. Although, a higher proportion of infants with mothers with PHIV had birth defects in our study, this difference was not statistically significant and based on small numbers.

Of note, the large majority of mothers with PHIV in the United Kingdom were born prior to 1994 when antiretroviral prophylaxis for preventing mother-to-child HIV transmission started being used. It will be important to continue to monitor the long-term health of HEU infants born to women with PHIV.

Ideally, the comparison group would also have been matched to the PHIV group by country of birth, but low numbers of BHIV women with younger age at first conception precluded this. Although the study does collect ’baseline’ viral load data from the beginning of pregnancy, there was a considerable proportion of missing data so we were unable to include it in our analysis. There may have been under-reporting of pregnancies ending in early termination or miscarriage that did not come to the attention of our respondents in antenatal care settings for both BHIV and PHIV women. Thus, the incidence of first pregnancy reported here is a minimum estimate and median age at first conception could be lower than our estimate. We were unable to calculate an incidence of first pregnancy in BHIV women as the study does not collect data on women who do not become pregnant.

As previously noted, differences in case ascertainment may exist between the two groups, as women with PHIV may be more closely monitored. As we do not routinely collect information on smoking, other substance use, hypertension, prior treatment history, or measures of adherence, we were unable to account for these factors. The sample size limitations in our study mean that differences in low prevalence adverse birth outcomes may not have been detected. In terms of generalizability, our data are national and therefore representative.

As cohorts of women with PHIV continue to age, and as younger women and adolescents with PHIV have accessed better treatment and therefore have improved health status, the incidence of planned pregnancy in women with PHIV is likely to rise, in keeping with high rates of procreational intent reported in young people with PHIV in the United Kingdom [31]. However, the current cohort of adolescents with PHIV have reported low rates of sexual activity; if recent work on improving transition from paediatric to adult services increases access to and use of effective contraception [38], incidence of unplanned pregnancy in this group may fall. It is imperative that the complex health needs of women with PHIV before, during, and after pregnancy are recognized and addressed. We have demonstrated that women with PHIV in the United Kingdom are less likely to achieve an undetectable viral load in pregnancy, but further work on larger cohorts is required to establish the effects of perinatal HIV infection on fertility and on pregnancy/birth outcomes in the current treatment era. Our findings confirm a need to closely follow and support this group of women living with PHIV in their reproductive years and beyond, as their health status and often complex treatment profile have implications for a second generation of perinatally infected and HEU children.

## Acknowledgements

C.T., C.F., and P.A.T. provided initial conceptualisation. L.B. conducted the statistical analyses with the support of C.T., P.A.T., and M.C-B. and drafted the paper. R.S. and C.T. participated in further developing the concept of the paper and interpretation of the results. All authors commented on drafts of the paper and approved the final version.

The national surveillance of obstetric and paediatric HIV is undertaken through the National Study of HIV in Pregnancy and Childhood (NSHPC), in collaboration with Public Health England and Health Protection Scotland. The authors gratefully acknowledge the contribution of the midwives, obstetricians, genitourinary physicians, paediatricians, clinical nurse specialists, and all other colleagues who report to the NSHPC through the British Paediatric Surveillance Unit of the Royal College of Paediatrics and Child Health and the obstetric reporting scheme run under the auspices of the Royal College of Obstetricians and Gynaecologists. We wish to thank Icina Shakes (former Study Assistant), Anna Horn (Study Assistant), Helen Peters (Study Co-ordinator/Statistician), and Kate Francis (Research Assistant) for their essential contributions to the NSHPC.

The National Study of HIV in Pregnancy and Childhood receives funding from Public Health England, including the National Health Service Infectious Diseases in Pregnancy Screening Programme. At the time of writing, L.B. held an MRC Clinical Research Training Fellowship (grant no. MR/J013706/1).

The UCL Great Ormond Street Institute of Child Health receives a proportion of funding from the Department of Health's National Institute for Health Research Biomedical Research Centres funding scheme.

### Conflicts of interest

There are no conflicts of interest.

## Figures and Tables

**Table 1:** Baseline characteristics of 118 women with behaviourally acquired HIV and 45 women with perinatally acquired HIV.

	Women with BHIV (*N* = 118)		Women with PHIV (*N* = 45)		
	*n*	%	*n*	%	*P* value
Ethnic group					0.43
White	37	32	11	24	
Black African	67	57	26	58	
Other	13	11	8	18	
Region of birth					0.02
UK/Ireland	41	36	26	58	
Africa	62	54	18	40	
Elsewhere	12	10	1	2	
Injecting drug use^a^	3	3	0	0	0.28
Median age at diagnosis (years; IQR)	19.1 (17.4, 20.5)		5.6 (2.7, 11.1)		<0.01
Median age at first conception (years; IQR)	20.1 (18.8, 23.0)		19.8 (17.7, 21.4)		0.02
Age at first conception (years, grouped)					–
<16	0	0	3	7	
16–19	64	54	24	53	
20–24	48	41	16	36	
25–29	6	5	2	4	

BHIV, behaviourally acquired HIV; IQR, interquartile range; PHIV, perinatally acquired HIV.

^a^Women with injecting drug use as likely route of acquisition of HIV.

**Table 2:** Clinical factors and pregnancy outcomes in women with perinatally acquired HIV and behaviourally acquired HIV.

	Pregnancies in women with BHIV (*N* = 184)		Pregnancies in women with PHIV (*N* = 70)		
	*n*	%	*n*	%	*P* value
On ART at conception	(*n* = 181)		(*n* = 69)		
	69	39	45	65	<0.01
Type of antenatal ART	(*n* = 178)		(*n* = 69)		
None	5	3	4	6	0.32
Monotherapy	4	2	0	0	
cART	169	95	65	94	
Baseline CD4^+^ cell count in pregnancy (cells/μl)	(*n* = 171)		(*n* = 66)		
≥500	63	37	21	32	<0.01
350–499	54	31	9	14	
200–349	44	26	22	33	
<200	10	6	14	21	
Outcome of pregnancy	(*n* = 184)		(*n* = 70)		
Live birth	163	88.5	58^a^	83	0.02
Stillbirth	1	0.5	0	0	
Miscarriage	12	6	2	3	
Termination	5	3	9	13	
Continuing	3	1	1	1	
Median weeks gestation at booking^b^, (IQR)	(*n* = 115)12.1 (10.3, 15.4)		(*n* = 57)11.6 (9.4, 13.9)		
Mode of delivery^c^	(*n* = 163)		(*n* = 56)		
Vaginal	57	35	18	32	0.88
Elective caesarean section	61	37	23	41	
Emergency caesarean section	45	24	15	27	
Preterm delivery^c^	(*n* = 163)		(*n* = 56)		
<37 weeks gestation	20	12	9	16	0.47
≥37 weeks gestation	143	88	47	84	
Low birth weight in term infants^c^	(*n* = 139)		(*n* = 46)		
<2500 g	12	9	3	7	0.65
≥2500 g	127	91	43	93	

NB, lower than expected denominators indicate missing data. BHIV, behaviourally acquired HIV; cART, combined antiretroviral therapy; IQR, interquartile range; PHIV, perinatally acquired HIV.

^a^There was one twin pregnancy in the PHIV group ending in live birth, so total 59 infants were born in this group.

^b^Pregnancies ending in live birth or continuing to term only.

^c^Pregnancies ending in live birth.

**Table 3:** Risk factors for having a detectable viral load near to delivery in live births.

Explanatory variable	Viral load >50 copies/ml	Invariable analysis			Multivariable analysis (*n* = 206)		
	*N* (%)	OR	95% CI	*P* value	aOR	95% CI	*P* value
Maternal HIV acquisition							
BHIV	32/158 (20)	1			1		
PHIV	22/55 (40)	2.63	1.24–5.55	0.01^a^	3.22	1.22–8.48	0.02^a^
Age at first conception (continuous/year older)		0.83	0.73–0.95	<0.01^a^	–	–	–
Parity							
Nulliparous	37/127 (29)	1			–	–	–
Multiparous	17/86 (20)	0.41	0.34–1.07	0.08	–	–	–
Previous AIDS-defining illness^b^							
No	22/101 (22)	1			–	–	–
Yes	7/12 (58)	5.03	1.32–19.17	0.02^a^	–	–	–
Age at conception (continuous/year older)		0.80	0.71–0.90	<0.01^a^	0.89	0.78–0.99	0.04^a^
Maternal region of birth							
UK/Ireland/Europe	26/89 (29)	1			–	–	–
Africa/Elsewhere	28/121 (23)	0.73	0.36–1.47	0.38	–	–	–
CD4^+^ cell count near conception							
≥500 cells/μl	13/80 (16)	1			1		
200–499 cells/μl	29/111 (26)	1.82	0.97–3.82	0.11	1.97	0.86–4.51	0.11
<200 cells/μl	10/19 (53)	5.73	1.59–20.61	0.01^a^	3.49	1.00–12.10	0.05
On ART at conception							
No	40/113 (35)	1			1		
Yes	13/99 (13)	0.28	0.12–0.62	<0.01^a^	0.27	0.11–0.70	<0.01^a^
PI-containing ART							
No	4/48 (8)	1			1		
Yes	48/161 (30)	4.67	1.64–13.34	<0.01^a^	3.52	1.16–10.69	0.03^a^
Gestational age at delivery							
≥37 weeks	44/184 (24)	1			–		
<37 weeks	10/29 (34)	1.67	0.76–3.70	0.20	–	–	–
Year of delivery							
2006–2008	18/63 (29)	1			–	–	–
2009–2011	21/84 (25)	0.83	0.40–1.75	0.63	–	–	–
2012–2014	15/66 (23)	0.74	0.33–1.63	0.45	–	–	–

aOR, adjusted odds ratio; BHIV, behaviourally acquired HIV; CI, confidence interval; OR, odds ratio; PHIV, perinatally acquired HIV; PI, protease inhibitor.

^a^*P* value reaches the level of significance (<0.05).

^b^The variable ‘previous AIDS-defining illness’ has a high proportion of missing data and so was not included in the multivariable analysis.
